# Prescribing efficiency of proton pump inhibitors in China: influence and future directions

**DOI:** 10.1186/s12913-014-0638-6

**Published:** 2015-01-22

**Authors:** Wenjie Zeng, Alexander E Finlayson, Sushma Shankar, Winnie de Bruyn, Brian Godman

**Affiliations:** School of Management, Chongqing Jiaotong University, No.66 Xuefu Road, Nan’an District, Chongqing, 400074 China; Green Templeton College, University of Oxford, Oxford, UK; Nuffield Department of Surgical Sciences, John Radcliffe Hospital, University of Oxford, Headington, Oxford, OX3 9DU UK; Department of Pharmaceutical Sciences, Utrecht University, Utrecht, Netherlands; Department of Laboratory Medicine, Division of Clinical Pharmacology, Karolinska Institutet, Karolinska University Hospital Huddinge, Stockholm, SE-141 86 Sweden; Strathclyde Institute of Pharmacy and Biomedical Sciences, University of Strathclyde, Glasgow, UK; Liverpool Health Economics Centre, Liverpool University, Chatham Street, Liverpool, L69 7ZH UK

**Keywords:** Proton pump inhibitors, China, Drug utilisation, Generics, Health policies, Prices, Europe

## Abstract

**Background:**

Pharmaceutical expenditure is currently rising by 16% per annum in China, greater in recent years. Initiatives to moderate growth include drug pricing regulations, essential medicine lists and encouraging generic prescribing. These are principally concentrated in hospitals, which currently account for over 80% of total pharmaceutical expenditure. However, no monitoring of prescribing and perverse incentives encouraging physicians and hospitals to profit from drug procurement encourages irrational prescribing. This includes greater utilisation of originators versus generics as well as injectables when cheaper oral equivalents are available. The objective of the paper is to assess changes in proton pump inhibitor (PPI) utilisation and expenditure in China as more generics become available including injectables.

**Methods:**

Observational retrospective study of PPI utilisation and procured expenditure between 2004 and 2013 in the largest teaching hospital in Chongqing District as representative of China.

**Results:**

Appreciable increase in PPI utilisation during the study period rising 10.4 fold, with utilisation of generics rising faster than originators. Oral generics reached 84% of total oral preparations in 2013 (defined daily dose basis), with generic injectables 93% of total injectables by 2013. Injectables accounted for 42% of total PPI utilisations in 2008 and 2009 before falling to below 30%. Procured prices for oral preparations reduced over time (−34%). Generic oral omeprazole in 2010 was 87% below 2004 originator prices, mirroring reductions seen in Western Europe. Injectable prices also decreased over time (−19%). However, injectables typically 4.3 to 6.8 fold more expensive than equivalent orals - highest for injectable lansoprazole at 13.4 to 18.0 fold. High utilisation of more expensive oral PPIs as well as injectables meant that PPI expenditure increased 10.1 fold during the study period. Lower use of injectables, and only oral generic omeprazole, would result in accumulated savings of CNY249.65 million, reducing total accumulated expenditure by 84%.

**Conclusions:**

Encouraging to see high utilisation of generic PPIs and low prices for oral generics. However, considerable opportunities to enhance prescribing efficiency through greater use of oral generic omeprazole.

## Background

There is increasing scrutiny over pharmaceutical expenditure with growth rates averaging 50% in real terms among OECD countries during the past decade [1]. This is driven by well-known factors including changing demographics and new premium priced drugs [2,3]. This has resulted in a number of measures and initiatives across countries to moderate growth rates, including initiatives for both new and established medicines [4-7]. Reforms and initiatives for established drugs principally aim to enhance the utilisation of low cost generics versus originators and single sourced (patented) products in a class or related class where all products are seen as essentially therapeutically similar. Classes include the proton pump inhibitors (PPIs), statins and renin-angiotensin inhibitor drugs, with the latter including both angiotensin converting enzyme inhibitors (ACEIs) and angiotensin receptor blockers (ARBs) [7-12]. Efficiency savings can be substantial for these classes with prices of generics as low as 2% to 10% of pre-patent loss prices in some countries[13-15]. Considerable savings have also been achieved among low- and middle income countries from switching originators to the lowest-priced generic drugs [16-18].

China has also seen considerable increases in pharmaceutical expenditure, growing at over 16% per annum during the past decade and over 35% per annum in recent years [18-21]. This growth is attributable to several factors including urbanization, ageing populations, expansion in medical insurance, improvements in living standards and the irrational use of drugs [18]. China’s healthcare system has also experienced a transition from a planned economy to a market economy in recent years. Alongside this, the Chinese government has introduced different types of health insurance in recent years targeting different populations, with coverage reaching over 90% of the population by 2011 [20,22-24] although large disparities still exist [25]. The ultimate goal of the authorities in China is universal coverage by 2020 [22-24,26]. As a result of increased coverage, healthcare expenditure increased from 3.5% to 5% of GDP between 1995 and 2010, equating to a ten-fold increase in yearly per capita spending from US$ 21 to 220 [24]. This further increased to US$350 per year in 2011 [21]. Alongside this, the financial support from the Chinese government to public hospitals declined steadily in recent years from approximately 60% of hospital revenues in 1980s to 8.2% by 2003. As a result, pharmaceutical expenditure in hospitals now accounts for approximately 40% to 50% of their total income [18,21,27-30], with hospitals necessarily using the revenue from drug dispensing for their sustainability [20,24,28,31]. This has caused much concern in China regarding difficulties with obtaining medical services and their high cost.

Consequently, the authorities in China have introduced a number of measures in recent years to help moderate this growth in an attempt to reduce patients’ out-of-pocket burden expenditure especially for pharmaceuticals [18,19,24,25,32]. To date, these have principally concentrated on pharmaceutical prices and expenditure in hospitals since more than 80% of total pharmaceutical consumption is currently dispensed in public hospitals in China [18,19,33]. For state-priced products, i.e. mainly prescription medicines in the national medical insurance catalogue, the National Development and Reform Commission (NDRC) sets maximum retail prices (price caps); for province- or municipality-priced products (OTC in the national medical insurance catalogue or medicines supplemented in the local insurance catalogue), the price management department determines the retail prices; and for all other products the retail prices are determined by the manufacturers themselves [18,24,28,34]. For instance, the NDRC has implemented 28 price adjustments between 1997 and 2011 [18,20,35,36]. Medicines in hospitals are subsequently subject to tenders in each province and municipality, with each hospital pharmacy having its own product list. This tendering process is typically organized by the health administrative department of the provincial government as well as for non-profit medical institutions at or above the county level. The tendering procurement cycle is typically once yearly and the winner is a mixture of those with higher quality, lower price or a mixture of these. Published studies have suggested these bidding processes reduced prices of essential medicines by 16.9% between 2009 and 2011 [24]. However, there are no pricing policies for generics in China unlike measures across Europe, which has led to low prices in a number of countries[14,15,37-40].

Demand-side measures to contain pharmaceutical expenditure in China’s hospitals include the development of an essential medicine list, clinical guidance and guidelines to enhance the rational use of medicines [19-21,24,29,41,42]. There were reforms in 2007 - the ‘Prescription Management Ordinance’ - specifying that prescriptions should be written by INN. However, to date there has been limited enforcement [18,28]. As a result, physicians still tend to write prescriptions with the generic (INN) name and simultaneously indicate the brand or manufacturer name; alternatively, drugs are listed with the corresponding brand name or manufacturer in hospitals’ IT system [18,43].

However, the current incentive system, as well as limited demand-side measures, have resulted in considerable irrationality in prescribing despite measures to improve this [24,28,34]. This is illustrated by continued appreciable use of injectable drugs in China when oral tablets are available as alternatives without the potential for serious complications, e.g. antibiotics and steroids [31,34,36,44], greater prescribing of more expensive antibiotics [45] as well as considerable prescribing of traditional Chinese medicines (TCMs) with limited data on their effectiveness and safety [46].

PPIs are seen as standard treatment for a number of conditions including symptomatic treatment of gastro-oesophageal reflux disease (GORD), Helicobacter pylori infections and associated peptic ulcer disease as well as the management of peptic ulcer bleeding and the prevention of recurrent bleeding from peptic ulceration [47]. PPIs are also available as both oral tablets and intravenous injections (IV) in China. Oral therapy is seen as highly effective [48], similar in effectiveness to IV PPIs at equivalent doses [49]. However, we are aware that there may be considerable use of injectable PPIs in China and that generic PPIs will be available at lower cost than originators. Consequently, the rationale for evaluating the PPIs in China is included in Table 1.

**Table 1:** **Rationale for the studying PPIs in China [** 13 **,** 14 **,** 20 **,** 21 **,** 36 **,** 47 **,** 50 **,** 51 **]**

Key Factors	• The utilisation of antacids and medicines to prevent and treat ulcers has increased rapidly in recent years in China due to their effectiveness, similar to other countries
	• There is no appreciable difference therapeutically between the various PPIs, and between originator and generic PPIs (provided bioequivalence has been demonstrated)
	• Between 2004 and 2013, a range of generic PPIs were included in hospital lists in China with a considerable number available for potential procurement
	• Injectable PPIs (originator and generic) are also available at considerably higher costs than oral equivalents, and limited medical justification for their routine use
Opportunities	• to evaluate generic penetration rates and savings versus originators for both oral tablets and injectables
	• to compare and contrast PPI utilisation and expenditure patterns in China with those seen among Western European countries, which already provide universal and comprehensive healthcare and where multiple policies have been successfully introduced to enhance the prescribing of low cost oral generic tablets versus originators or patented (single-sourced) PPIs. In the Netherlands, combined measures resulted in expenditure for PPIs in 2010 58% below 2000 levels despite a 3-fold increase in utilisation, and in Scotland multiple measures resulted in expenditure on PPIs in 2010 56% below 2001 despite also a 3-fold increase in utilisation

Consequently, the principal objective of this study is to assess changes in PPI utilisation and expenditure in China as more generic PPIs are incorporated into hospital procurement lists including injectable and orals. Secondary objectives include assessing price reductions for the PPIs over time, and comparing the findings with other generics in China as well as Europe. Subsequently, suggesting potential future measures that the authorities in China could consider as they strive for universal access. This will be based on the experiences in Europe as China continues to strive for universal coverage.

## Methods

This was an observational retrospective study of prescriptions dispensed over a ten year period between 2004 and 2013 [52]. This methodology was chosen since multiple supply- and demand-side measures have been introduced during this period in China, some of which have been described in the Background, making it difficult to perform an interrupted time series analysis. In addition, an appreciable number of generic PPIs are now available in China, procured at different times.

Typically for these types of drug utilisation analysis, data is obtained from health authority, health insurance or pharmacy databases [7,11,50,53,54]. However in China, most drug utilisation studies are performed with data from hospitals, including urban healthcare facilities with in-patient beds, as they incorporate both inpatient and outpatient data [18,31,55]. In addition, as mentioned, they account for 80% of total drugs currently dispensed in China [18,33]. This is in view of the convenience of hospital dispensing, physician recommendations, possibility of nonstandardized prescriptions and greater assurance of pharmaceutical quality in hospitals [28]. Consequently, hospital procurement data is currently the most appropriate source of drug utilisation and expenditure data in China [46].

Chongqing is a municipality directly under China’s central government, with a total population of 28.8 million people (2010 census). In the urban district in Chongqing City, the main public general hospitals include three hospitals affiliated to the Third Military Medical University, two hospitals affiliated to Chongqing Medical University, and 10 municipal hospitals. Every hospital may include different generic drugs from different manufacturers, but with the same originator equivalents as there are only a limited number of originator manufacturers [18,56,57].

In view of these factors, we chose the largest hospital in Chongqing District to conduct our study. This is because it is one of the largest hospitals in Southwest China and is a typical health provider to the public, has a wide range of medicines available for prescribing, and can provide comprehensive datasets on both utilisation and expenditure. The dataset was obtained from the magazine company of *China Pharmacy*. The company is located in Chongqing and is able to collect detailed information of drug procurement from large hospitals in southwest China through co-operation with these public hospitals. The data contains all individual procurement information including product names, purchase dates, dosage forms, specifications, manufacturers, unit prices and volumes. This is an authoritative source for drug utilisation statistics in China, which is regularly audited [18,46,56].

Six PPIs were available for analysis between 2004 and 2013. These were omeprazole, lansoprazole, pantoprazole, rabeprazole, esomeprazole, and ilaprazole (ATC C09CA01 to 09, C09DA01 to 05, C09DX01 to 03) [58]. Originator and generic PPIs were procured at different times with, as mentioned, an appreciable number of generics typically available for procurement. Originator PPIs are referred to as products currently or previously possessing intellectual property (patent), most of which are manufactured by joint ventures in China founded by global pharmaceutical companies. Generic drugs are those produced by Chinese enterprises with local investment. Utilisation was measured in terms of Defined Daily Dose, with DDDs defined as ‘*the average maintenance dose of a drug when used in its major indication in adults*’, with this measure recognised as the international standard to assess utilisation patterns within and between countries [59]. 2012 DDDs were used in line with international guidance [59-61].

The Chinese currency Renminbi “*yuan*” (CNY) was used to determine expenditure and expenditure/DDD for PPIs over time. These were not adjusted for inflation or deflation during this period as we wanted to compute actual changes over time as a result of the tendering process. This mirrors similar studies across Europe, especially where the tendency of authorities is to reduce medicine prices to keep pharmaceutical expenditure under control [5,7,39,50,53]. We have also not converted CNY data to either US$ or Euros during the course of the study as we did not want the pricing data influenced by currency fluctuations especially during the recent financial crises in Europe and the US.

## Results

Utilisation (DDDs basis) and expenditure (CNY) were analysed over time including both generics and originators for both oral and injectable PPIs.

### Utilisation

**Figure 1:**
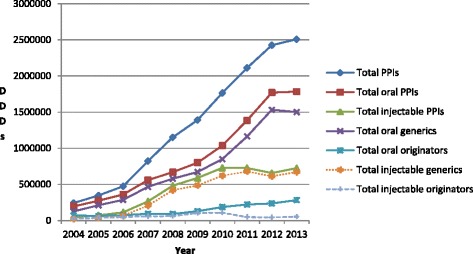
**Utilisation of oral and injectable PPIs (in DDDs) between 2004 and 2013 in the Chongqing hospital.**

There was an appreciable increase in the prescribing of PPIs, rising 10.4 fold from just over 242,000 DDDs in 2004 to 2.51 million in 2013 (Figure 1). The greatest increase (15.7 fold) was seen with the injectable PPIs. At one stage (2008 and 2009), injectable PPIs accounted for 42% of total PPI utilisation before falling to below 30% in recent years (Figure 1).

Utilisation of oral generic PPIs grew at a faster rate than oral originators (Figure 1). As a result, the % of total oral generic PPIs grew from 64% in 2004 (DDD basis) to between 82% and 87% between 2007 and 2013. There was also greater growth for generic versus originator injectable PPIs, growing from 46% of total injectables in 2004 to 93% between 2011 and 2013 (DDD based).

**Figure 2:**
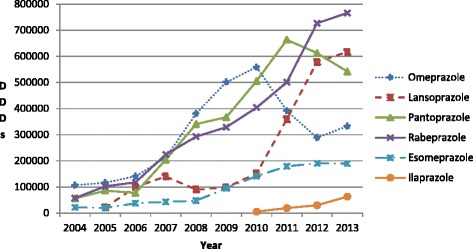
**Total utilisation of the different PPIs (all formulations) in the Chongqing hospital from 2004 to 2013.**

The utilisation of all forms of lansoprazole (originator and generic, oral and injectable) grew 28.4 fold during the course of the study compared with 13.3 fold for rabeprazole, 9.7 fold for pantoprazole and 3.1 fold for omeprazole. The growth of lansoprazole was especially strong after the launch of generic injectable lansoprazole in 2010 (Figure 2). The decline in the utilisation of pantoprazole from 2010 onwards was due to falling utilisation of generic injectable pantoprazole. Generic omeprazole had not been procured since July 2010, with the utilisation of generic injectable omeprazole declining from 2010 onwards. Both factors resulted in the lower utilisation of omeprazole in recent years (Figure 2).

### Expenditure

**Figure 3:**
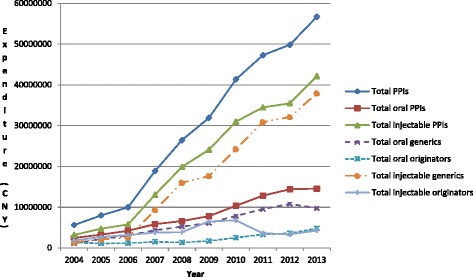
**Expenditure (CNY) of oral and injectable PPIs between 2004 and 2013 in the Chongqing hospital.**

Total PPI expenditure increased steadily during the study period, rising 10.1 fold from just over 5.6 million CNY in 2004 to 56.7million CNY in 2013. Growth in expenditure on injectable PPIs was greater than for oral PPIs, with expenditure on injectable PPIs increasing from 57% of total injectable PPI expenditure in 2004 to 71% to 74% between 2008 and 2013 (Figure 3). There was a variable contribution of generic oral PPIs to total oral PPI expenditure (Figure 3), reaching a maximum of 80% in 2008. However, there was steady growth in the contribution of generic injectable PPIs to total expenditure on injectable PPIs, reaching between 90% and 91% of total injectable PPI expenditure between 2011 and 2013 (Figure 3).

**Figure 4:**
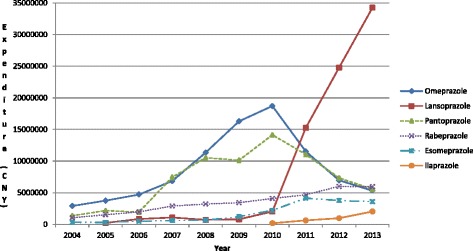
**Total expenditure (CNY) of the different PPIs (all formulations) in the Chongqing hospital from 2004 to 2013.**

The greatest increase in expenditure was observed with lansoprazole at approximately 160 fold, least for omeprazole (1.8 fold) (Figure 4). This reflects increased utilisation of injectable lansoprazole in recent years (Figure 2), with injectable PPIs overall typically 4.2 to 6.8 fold more expensive (CNY/DDD) than their equivalent oral formulations (Table 2). This difference is greater for injectable lansoprazole at 13.4 to 18.0 fold higher than the equivalent oral formulation between 2010 and 2013 (Table 2).

**Table 2:** **Procured expenditure/DDD (CNY) for the different PPIs (generic and originator) in the Chongqing hospital from 2004 to 2013**

**PPIs**	**Dosage form**	**2004**	**2005**	**2006**	**2007**	**2008**	**2009**	**2010**	**2011**	**2012**	**2013**	**% change overtime**
Omeprazole Generic	Oral	4.15	4.11	3.42	3.27	3.27	3.03	1.85				−55%
Omeprazole Generic	Injection	38.89	34.29	27.16	27.87	27.25	28.09	29.75	29.41	22.91	14.50	−63%
Omeprazole Originator	Oral	14.19	13.87	12.16	12.11	11.86	11.85	11.70	11.58	11.85	11.83	−17%
Omeprazole Originator	Injection	73.21	70.61	65.30	64.65	62.18	62.18	61.28	60.77	56.42	45.32	−38%
Lansoprazole Generic	Oral		9.92	8.33	7.69	7.79	8.10	8.21	6.88	5.74	5.17	
Lansoprazole Generic	Injection							110.00	104.11	96.30	93.04	
Pantoprazole Generic	Oral	9.86	9.68	8.91	8.44	8.64	8.81	8.83	7.54	6.10	5.22	−47%
Pantoprazole Generic	Injection	92.15	76.74	63.24	58.68	55.31	54.05	53.72	34.13	27.51	21.68	−76%
Pantoprazole Originator	Injection									109.67	108.69	
Rabeprazole Generic	Oral	11.99	12.74	15.28	11.50	10.29	10.23	10.11	9.32	8.25	7.78	−35%
Rabeprazole Originator	Oral	33.94	33.15	30.59	31.50	30.37	30.37					−100%
Esomeprazole Originator	Oral	14.08	15.20	13.23	13.72	13.73	12.88	13.20	13.60	12.80	12.64	−10%
Esomeprazole Originator	Injection					93.00	93.00	93.00	93.00	91.65	90.65	
Ilaprazole Originator	Oral							32.83	32.34	32.22	32.22	
Total oral		12.35	11.91	11.60	10.43	9.84	9.74	9.98	9.24	8.12	8.16	−34%
Total injectable		68.81	64.56	50.97	49.18	41.22	40.84	42.54	47.39	53.07	55.40	−19%
Differences between injectable and oral (fold)		5.6	5.4	4.4	4.7	4.2	4.2	4.3	5.1	6.5	6.8	

NB - % change over time typically refers to 2013 vs. 2004. However, this can vary depending when the different formulations were first procured. Blank cells mean no PPI available. Fold = times, e.g. total injectable PPIs in 2004 were 5.6 times more expensive per item than the equivalent oral preparations (DDD based).

There were price reductions for the various PPI formulations over time (Table 2). Overall, price reductions were greater for the oral formulations combined (−34%) than for the injectable formulations combined (−19%). However, the percentage reduction over time was different between the different PPIs as well as their different formulations (Table 2). Generally the procured price reductions were greater for generic formulations of the various PPIs than for the originator formulations (Table 2).

The procured price for generic oral omeprazole in 2010 was 87% below the originator price in 2004 (expenditure/DDD), with the price of generic injectable omeprazole in 2013 80% below the 2004 originator price. Generic oral rabeprazole in 2013 was 77% below 2004 originator oral rabeprazole (Table 2).

## Discussion

There was appreciable growth in the utilisation of both oral and injectable PPIs in the Chongqing district between 2004 and 2013, similar to the appreciable increase in the utilisation of oral PPIs among Western European countries during this period [13,14,39,50].

It was encouraging to see appreciable utilisation of generic oral PPIs, averaging over 80% of total PPIs on a DDD basis since 2007 (Figure 1). This is similar to the high rate of utilisation of generic vs. originator and patented (single-sourced) PPIs in the Netherlands, Scotland and Sweden [13,14,50]. The high utilisation of oral generic PPIs is enhanced by some originator companies not being part of hospital procurement process and/or withdrawing from the hospital procurement, e.g. lansoprazole or pantoprazole (Table 2). However, there was no generic esomeprazole or ilaprazole during the study period (Table 2). This is in marked contrast to the situation seen with low utilisation of oral generic drugs for cardiovascular diseases in the Chongqing District including the ARBs and statins [18,46,56].

We believe these differences between the various product classes could be attributable to a number of reasons. Firstly, diseases of the cardiovascular system are seen as having greater importance in China compared with acid-related stomach disorders, and originator medicines are thought to have a more consistent effect in treating cardiovascular diseases than generics. Secondly, there are few local traditional Chinese medicines to treat peptic ulcer diseases unlike cardiovascular and cerebrovascular diseases [46]; consequently, domestic generic oral manufacturers have less competition. Lastly, it is believed some physicians think that doubling the dose of oral generic PPIs could lead to the same effectiveness as the originators at the standard dose, and this is acceptable in this situation. This contrasts with concerns among physicians with doubling doses for pharmacological treatments for cardiovascular diseases. However, we cannot say this with certainty without further research.

It was also encouraging to see the prices of generic oral PPIs reduce appreciably over time (Table 2). This is similar to the situation for generic simvastatin in China [57] as well as among Western European countries including the Netherlands, Sweden and the UK [13-15]. In addition, the procured price of generic omeprazole in 2010 was 87% below the 2004 originator procurement prices, matching the price reductions seen among Western European countries for generic omeprazole [13-15] as well as generic simvastatin in China [57].

However, there was variable use of the different oral and injectable PPIs suggesting continued irrationality in prescribing. The most utilised PPI was rabeprazole (DDDs basis), which had the highest expenditure/DDD for both the originator (when procured) and the generic versus the other oral PPIs (Table 2). In addition, generic oral omeprazole disappeared from the procurement list since July 2010 and at the time of its disappearance it had the lowest procured price (Table 2). There was also growing use of premium priced esomeprazole and ilaprazole, once available (Figure 2 and Table 2).

Furthermore, there was appreciable utilisation of injectable PPIs (Figure 2), reaching a maximum of 41% to 42% of total PPI utilisation (DDD based) between 2008 and 2010 before reducing to under 30% in 2013. This utilisation is considerably higher than the WHO guidelines for injections among developing countries [36,44], and appreciably higher than suggested limited use generally given the effectiveness of oral PPIs [49]. We believe this high utilisation is driven by considerably higher expenditure for injectable versus oral PPIs, averaging 4.3 to 6.8 fold or greater in recent years, especially lansoprazole injectable (Table 2), given the pressure on hospitals and physicians to make money from drug dispensing discussed earlier. This hypothesis is endorsed by a recent study which showed more appropriate use of injectables in China versus oral tablets, in line with WHO recommendations, once the procurement incentives had been removed coupled with programmes to enhance the rational use of medicines [36].

Consequently, there appears to be considerable opportunities to enhance the efficient use of PPIs in China to conserve resources without compromising care. This includes enhancing the utilisation of low cost oral generics versus originators and existing single sourced products, e.g. esomeprazole and ilaprazole, as well as oral versus injectable PPIs. Restricting hospital procurement to just one oral PPI, i.e. generic omeprazole, following similar initiatives among European countries and regions, e.g. the ‘Wise List’ in Stockholm Metropolitan Healthcare Region [62,63], and assuming its procured price in 2010 continued to the end of the study (Table 2) as well as limiting the utilisation of injectable generic PPIs to just 5% of total PPIs – generic omeprazole (cheapest) - at its procured price each year, would have saved an accumulated estimated CNY249.65 million for this hospital during the study period. This amounts to 84% of total accumulated PPI expenditure.

However, future demand-side measures are likely to have only limited success unless the current incentives encouraging physicians and hospitals to prescribe and dispense premium priced products including injectables are addressed. This is already happening as seen with recent initiatives among rural populations and public primary care providers in China to improve patient coverage, improve the provision of community health organisations, as well as enhance the rational use of medicines, which includes a 0% mark-up for public primary care providers [36,46,64]. In addition, pilot studies of different methods across China including remunerating providers, separating revenues from expenditures as seen among urban community health centers in Beijing, Chengdu and Hangzhou, collective bidding as well as implementing standard clinical treatment pathways [20]. These initiatives must continue. As a result helping to further reduce the utilization of injectable PPIs over time, building on recent changes.

Once these measures are underway, introducing concepts such as the ‘Wise List’ to limit the prescribing of medicines in classes and disease areas to those with the most robust data on effectiveness and safety as well as available prices [62] throughout the hospitals in the Chongqing District, along with continuous medical education and strengthening of hospital DTCs, should further enhance the quality and efficiency of prescribing [63]. This could provide an example to other provinces and municipalities throughout China as they grapple with similar issues to improve the rationality and efficiency of their prescribing. This though will require strong leadership to achieve this, including instigating quality measures and involving prescribers [65]; however, the potential economic benefits are considerable.

Finally, we acknowledge this research is subject to limitations. These include the fact that data collection was from just one region and one hospital. However, we believe these findings are generalizable to other drug classes and other hospitals in China based on the merits of our methodology as well as the realities of current regulations and tendering systems in China. We have also not looked at the appropriateness of prescribing for PPIs with growing concerns with their overprescribing [66]. There are also concerns with the side-effects of long-term use, i.e. an increase in infection rates including hospital and community-acquired pneumonia as well as osteoporosis, which can result in increased fracture rates [67-71]. However, this is difficult without access to the patient records.

## Conclusions

We believe we have demonstrated that despite recent measures there is still considerable irrationality in prescribing in China, especially around the high utilisation of injectable PPIs. There are also considerable opportunities to conserve resources without compromising care. Proposed measures include initiatives to enhance the rational use of medicines building on current pilot programmes as well as programmes among primary healthcare institutions in China.
